# Global research trends in m^6^A demethylases and cancer: a bibliometric and visualization analysis

**DOI:** 10.3389/fgene.2026.1943884

**Published:** 2026-10-07

**Authors:** Hao Lei, Hui Guo, Rong Lei, Jumei Sun, Yuanhang Yu, Lei Li, Chunping Liu, Guanghua Fu

**Affiliations:** 1 Department of General Surgery, The Eighth Hospital of Wuhan, Wuhan, China; 2 Department of Breast and Thyroid Surgery, Union Hospital, Tongji Medical College, Huazhong University of Science and Technology, Wuhan, China; 3 The Disinfection Supply Center of Xianfeng County People’s Hospital, Xianfeng, China; 4 Department of Cardiovascular Internal Medicine, Xianfeng County People’s Hospital, Xianfeng, China; 5 Department of Breast and Thyroid Surgery, The Second Affiliated Hospital of Hainan Medical University, Haikou, China

**Keywords:** bibliometric, citespace, m6A demethylase, tumor, VOSviewer

## Abstract

**Background:**

The role of m^6^A demethylases in tumorigenesis has garnered increasing attention; however, the global research landscape and thematic evolution in this field remain to be fully elucidated. This study aims to investigate the dynamic scientific development of the m^6^A demethylase-tumor field through bibliometric analysis.

**Methods:**

Literature pertaining to m^6^A demethylases and tumors published between 2015 and 2025 was retrieved from the Web of Science Core Collection (WoSCC) database. Quantitative and visual analyses were subsequently performed using the bibliometrix package in R. A cross-database sensitivity analysis using PubMed was conducted to assess whether the descriptive patterns were generally consistent across databases.

**Results:**

A total of 761 eligible publications were included in this study. The field has experienced rapid growth since 2020, with an average annual growth rate of 50.92%. China and the United States emerged as the leading contributors in terms of publication output. Research efforts were concentrated at institutions such as Sun Yat-sen University, forming a highly productive collaborative cluster centered on LI YANG. Keyword analysis revealed that gastric cancer, liver cancer, and colorectal cancer constituted the core cancer types examined. The research focus has evolved from early investigations of basic mechanisms to the tumor immune microenvironment, therapeutic resistance, and prognostic evaluation. The annual trend and geographical distribution obtained by PubMed sensitivity analysis are consistent with the direction of WoSCC results, suggesting that the main conclusions have not changed substantially because of database selection.

**Conclusion:**

Research in the m^6^A demethylase-tumor field is advancing rapidly. Current research efforts are predominantly directed toward the remodeling of the immunosuppressive tumor microenvironment by demethylases and the underlying mechanisms of therapeutic resistance. These findings provide a valuable direction and a novel perspective for further investigation of m^6^A demethylases in oncology.

## 1 Introduction

Over 170 distinct chemical modifications have been identified in RNA, which broadly regulate post-transcriptional RNA behavior and biological functions (Li et al., 2022). Among these, N6-methyladenosine (m6A) was first discovered in eukaryotic messenger RNA and viral nuclear RNA in the 1970s, and has since been established as one of the most prevalent and abundant RNA modifications (Desrosiers et al., 1974; Wang et al., 2020). m^6^A plays a pivotal role in regulating RNA splicing, translation, stability, and degradation (Sun et al., 2023). The dynamic equilibrium of m6A modification is orchestrated by three classes of proteins: writers (methyltransferases, e.g., METTL3/METTL14), erasers (demethylases, e.g., FTO and ALKBH5), and readers (m^6^A-binding proteins, e.g., the YTHDF family) (Zeng et al., 2020; Guan et al., 2022). Together, these regulators constitute a complex m^6^A regulatory network that influences diverse cellular physiological and pathological processes. Among the various RNA modifications, m^6^A has attracted considerable attention due to its extensive functional repertoire and dynamic reversibility. In particular, the discovery of m^6^A demethylases has revealed the dynamic and reversible nature of m^6^A modification in gene expression regulation. By reversing m^6^A modification, demethylases exert distinct regulatory functions in tumor biological behaviors, including tumor cell proliferation, resistance to apoptosis, invasion and metastasis, maintenance of cancer stemness, and remodeling of the tumor microenvironment.

Although substantial progress has been made in elucidating the biological functions and molecular mechanisms of m^6^A demethylases in cancer, the overall developmental trajectory, international collaboration landscape, distribution of core research institutions, and the evolution of scientific themes in this field remain to be systematically characterized. Traditional narrative reviews are often constrained by authors’ subjective preferences, making it difficult to provide a comprehensive and objective depiction of a discipline at a macro level. Bibliometrics, an interdisciplinary field grounded in mathematics and statistics, enables a systematic and impartial evaluation of knowledge domains. By mining latent information from large-scale literature datasets, it can objectively reveal the developmental trajectory, Frontier hotspots, and future trends within a specific research field. This study aims to delineate the key milestones in the development of m^6^A demethylase research in oncology and to identify current research hotspots and emerging trends. Through this panoramic analysis, we seek to provide researchers with insights into international collaboration, topic selection, and disciplinary integration, thereby facilitating the clinical translation of m^6^A demethylase-related findings and offering a new perspective for the innovation of cancer treatment strategies.

## 2 Materials and methods

### 2.1 Retrieval strategy

The Web of Science (WoS) is a comprehensive academic database platform and one of the most authoritative tools for literature retrieval and analysis worldwide. It encompasses high-quality academic literature across the natural sciences, social sciences, and arts and humanities, and provides robust bibliometric analysis capabilities. We retrieved relevant publications from the WoSCC database and established a dataset for bibliometric analysis. Detailed Medical Subject Headings (MeSH) terms for tumors are provided in Supplementary Table S1; notably, no MeSH term currently exists for m^6^A demethylase. To minimize the risk of misclassification and improve data purity, the 761 records found in the initial search were all filtered. Two reviewers (R1 and R2) independently reviewed the titles and abstracts of these 761 records. The records with differences are resolved by R1 and R2 through discussion; If no agreement can be reached, the third reviewer (R3) will make a decision. All the records that passed the title/abstract screening, as well as those whose qualifications were in doubt, were evaluated in full. After full-text evaluation, 761 records all meet the requirements. There is no so-called’ validation’ subset because no separate validation samples are used (Pitr et al., 2023).To prevent errors arising from database updates, all data were searched and exported on 25 August 2026. To minimize potential analytical bias, the following inclusion criteria were applied: (1) publications written in English; (2) document types limited to articles and reviews; and (3) publication date ranging from 1 January 2015 to 31 December 2025. After applying these criteria, a total of 761 publications were ultimately obtained for in-depth analysis. The detailed literature screening flowchart is shown in Figure 1 and Supplementary Table S2. In addition, the PubMed database was supplemented and cross-database sensitivity analysis was carried out. See Supplementary Table S2 for specific retrieval. Unlike WoSCC, PubMed offers more granular indexing of clinical study types and trial designs, enabling more precise identification of clinical research (Cheng et al., 2026).

**FIGURE 1:**
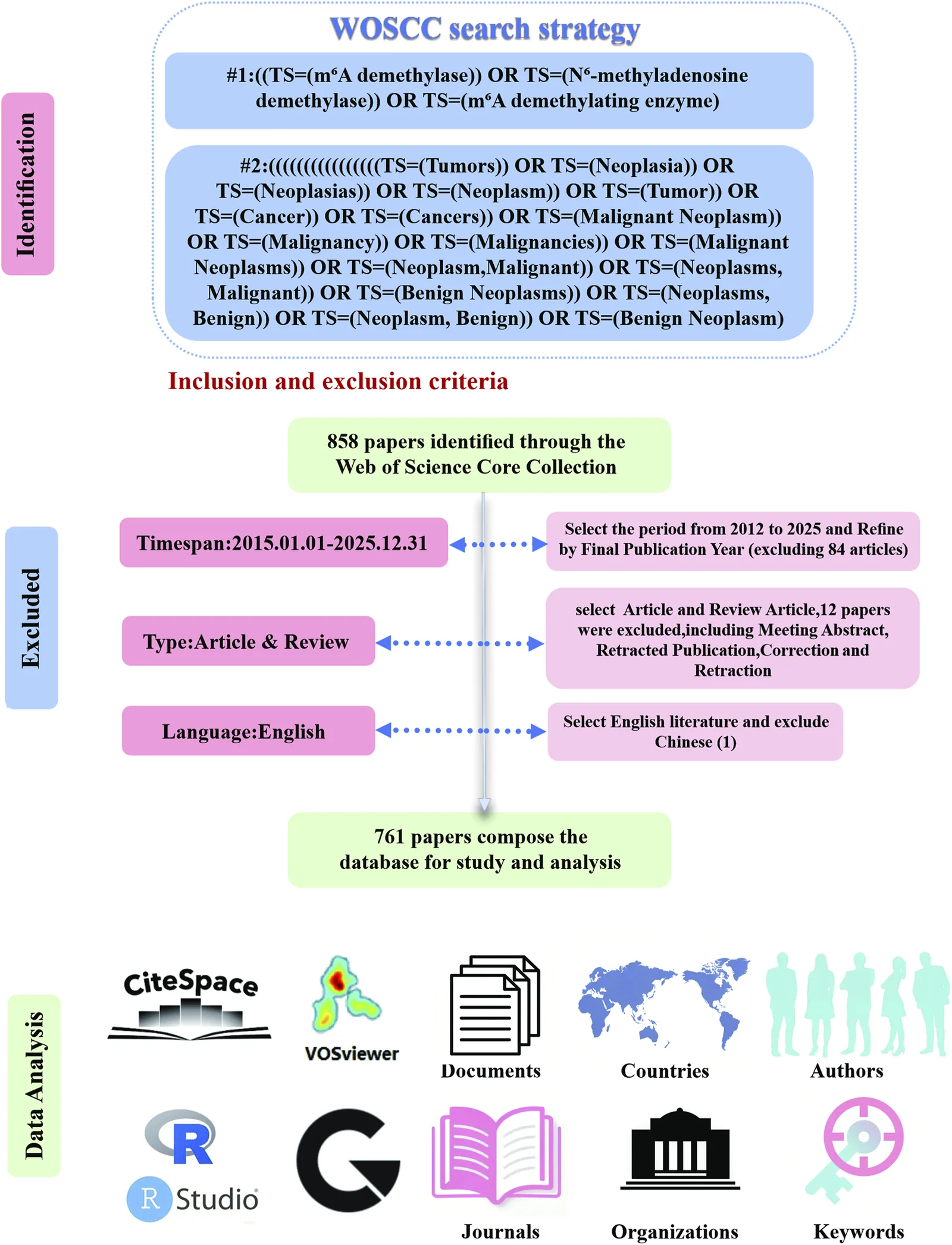
Research framework of m^6^A demethylases in the field of oncology.A systematic search in this field was conducted through WoSCC, and 761 eligible publications were obtained.

### 2.2 Data analysis

Multidimensional quantitative analyses were performed using established bibliometric tools. The bibliometrix package (version 4.6.1) for R is an open-source package developed by Massimo Aria and Corrado Cuccurullo for bibliometric analysis. It offers comprehensive functionalities to process and analyze literature data retrieved from major scientific databases (e.g., Web of Science, Scopus, PubMed) and to generate various statistical and visual outputs. Additional analyses were conducted using the online platform Bibliometric.com, Scimago Graphica, CiteSpace (version 6.4.R1), and VOSviewer (version 1.6.20). CiteSpace, a Java-based citation visualization software developed by Chaomei Chen, enables statistical analysis and transforms raw data into visual representations of literature networks (Chen, 2004); it was used to analyze keywords and references with significant burst strength. VOSviewer, a bibliometric mapping software developed by Nees Jan van Eck and Ludo Waltman in 2009 (van Eck and Waltman, 2010), was used to visualize co-authorship and institutional collaborations. By integrating the above software tools, we systematically analyzed the research field (Lei et al., 2025).

## 3 Results

### 3.1 Publishing trends and regional distribution characteristics

Ultimately, 761 publications pertaining to m^6^A demethylases and tumors were included in this study, comprising 538 articles and 223 reviews. This field is characterized by substantial interdisciplinary integration, with an average annual growth rate of 50.92% (Table 1). Over the study period, annual publication output exhibited a consistently upward trajectory. Notably, the literature output was limited between 2015 and 2019, whereas 709 publications appeared from 2020 onward, accounting for 93.2% of the total corpus, indicating that this field has emerged as a prominent research focus in recent years. Specifically, only two articles were published in 2015, the lowest annual output, while 2022 and 2023 each saw the highest output, with 137 articles published in each year (Figure 2A).

**TABLE 1:** General characteristics of the WoSCC database.Compound Annual Growth Rate (CAGR) calculated as: [(N_final/N_initial)^(1/years) - 1] × 100.

Description	Results
Timespan	2015:2025
Sources (journals, books, etc.)	310
Documents	761
Annual growth rate %	51.92
Document average age	3.57
Average citations per doc	55.57
References	22,896
Keywords plus (ID)	1120
Author’s keywords (DE)	1300
Authors	4160
Authors of single-authored docs	5
Single-authored docs	5
Co-authors per doc	8.29
International co-authorships %	14.32
Article	538
Review	223

**FIGURE 2:**
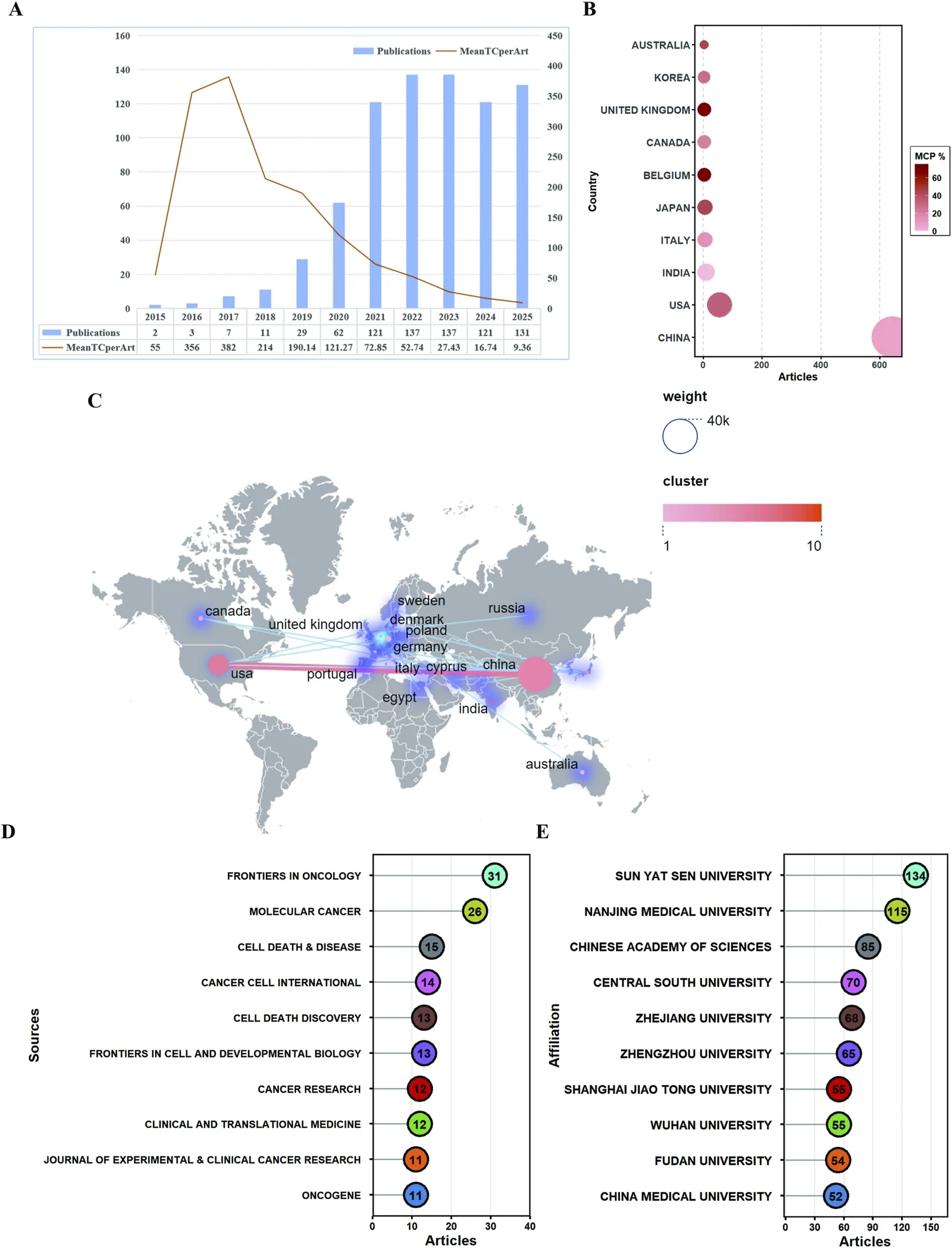
Publication trends and geographical distribution. **(A)** Annual publication output and citation metrics. **(B)** Bubble chart of the top ten countries by publication volume.The size of the bubble indicates the number of publications; MCP% represents the proportion of papers produced by international cooperation. **(C)** Global geographical distribution map.In the plain text file downloaded from WoSCC, “peoples r china” was replaced with “China” and “England” was replaced with “United Kingdom” to standardize country/region names. The updated file was then imported into VOSviewer to generate a GML format file, which was subsequently imported into Scimago Graphica for visualization. **(D)** Lollipop chart of the top ten most productive journals. **(E)** Lollipop chart of the top ten most productive institutions.

Twenty-seven countries or regions have contributed publications to the field of m^6^A demethylases and tumors. China ranked first with 644 articles, accounting for 84.63% of the total 761 publications. Among these Chinese articles, 578 were single-country publications (SCP), i.e., articles produced solely by authors from China without international collaboration, while 66 were multiple-country publications (MCP), i.e., articles co-authored by authors from multiple countries, representing 10.25% of China’s total output (Table 2). The global geographical distribution map further reveals that Asia, North America, and Europe are active research areas in this field, with close inter-regional collaboration (Figure 2B). Although China and the United States dominate in publication output, the United Kingdom (75%) and Belgium (75%) lead globally in terms of international collaboration activity, as measured by the proportion of MCP articles (Figure 2C).

**TABLE 2:** Countries/regions with Top10 posts and their collaboration rate.

Country	Articles	Articles %	SCP	MCP	MCP %
CHINA	644	84.63	578	66	10.25
UNITED STATES	55	7.23	34	21	38.18
INDIA	10	1.31	10	0	0.00
ITALY	6	0.79	5	1	16.67
JAPAN	6	0.79	3	3	50.00
BELGIUM	4	0.53	1	3	75.00
CANADA	4	0.53	3	1	25.00
UNITED KINGDOM	4	0.53	1	3	75.00
KOREA	3	0.39	2	1	33.33
AUSTRALIA	2	0.26	1	1	50.00

The top three journals in terms of publication output were Frontiers in Oncology (31 articles), Molecular Cancer (26 articles), and Cell Death and Disease (15 articles) (Figure 2D). These leading journals are classified as Q1 or Q2 in the Journal Citation Reports categories and are primarily situated within the fields of oncology and cell biology. The top three institutions with the highest publication output in this field were Sun Yat-sen University (134 articles), Nanjing Medical University (115 articles), and the Chinese Academy of Sciences (85 articles) (Figure 2E).

### 3.2 Research trajectory

The essence of a dual-map overlay lies in the relationship between the citing and cited domains, and the knowledge flow between disciplines at the journal level can be visualized through the overlay graph. In Figure 3, the left panel represents the primary distribution of citing journals in the field of m^6^A demethylase and tumor research from the WoSCC database, while the right panel shows the main distribution of cited journals. The research in this field is primarily concentrated in journal clusters related to molecular biology, immunology, clinical medicine, physics, and materials science. Analysis of the dual-map overlay reveals three distinct citation pathways in the knowledge dissemination within this field. The first pathway extends from molecular biology and immunology to molecular biology and genetics (yellow track), reflecting the continuity and deepening of knowledge within the discipline, as well as the accumulation of theoretical and mechanistic research. The second pathway extends from clinical medicine and neurology to drug toxicology and treatment (green track), embodying the translational medicine research paradigm driven by clinical questions. The third pathway originates from physics, materials, and chemistry to nursing and medicine (pink track), which highlights the interdisciplinary methodological innovations contributed by the physical sciences and materials engineering to this field, demonstrating the momentum of multidisciplinary collaboration.

**FIGURE 3:**
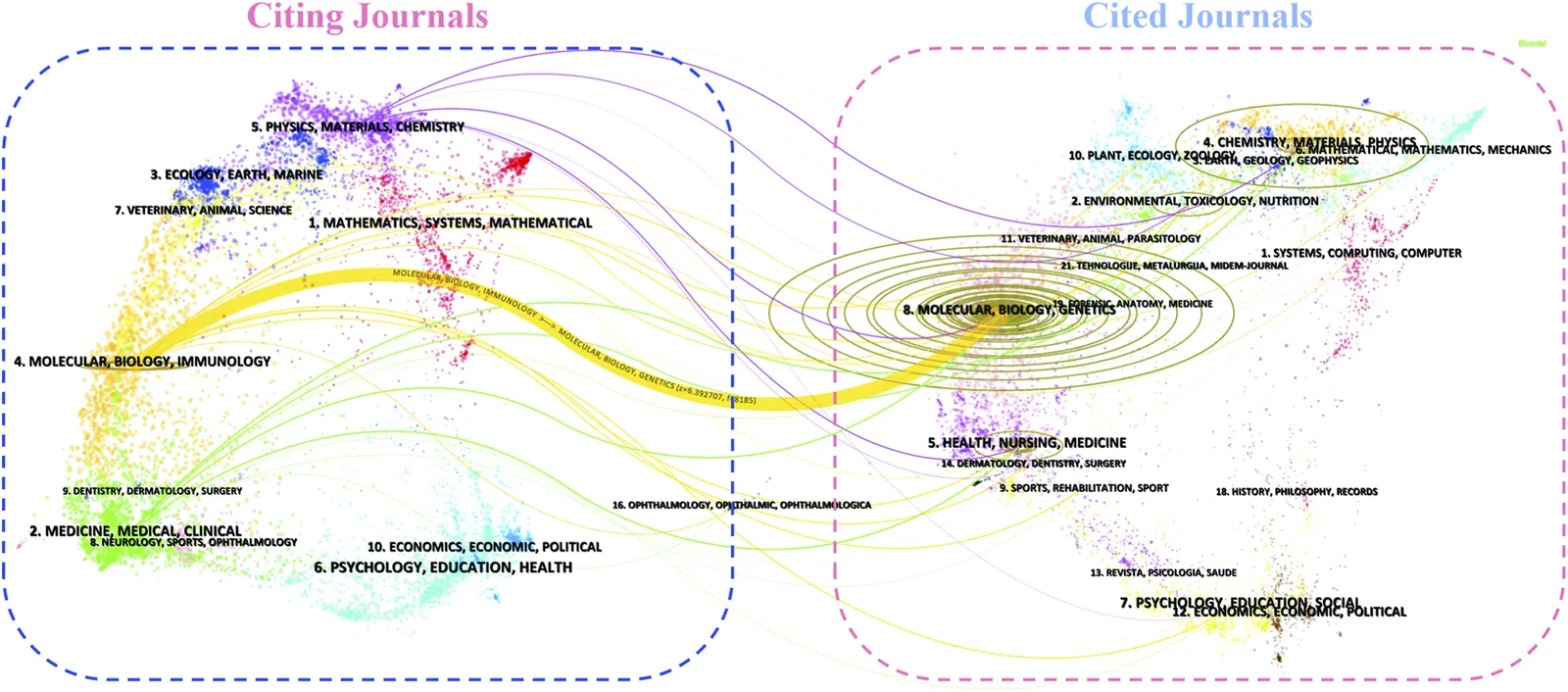
Double-map overlay analysis.The citing journals are displayed on the left and the cited journals on the right; colored tracks represent interdisciplinary citation paths. The thickness of the edges reflects the relative strength of the citation links.

### 3.3 Analysis on the annual publishing output of authors, journals and institutions

From 2015 to 2025, a total of 4,160 scholars worldwide contributed to the field of m6A demethylase and tumor research. According to Lotka’s law, 3,140 scholars (75.5% of the total) published only one paper, while 607 scholars (14.6%) published two or more articles. This distribution indicates a large but highly mobile research community, suggesting that the field remains in a relatively early stage of development, with overall productivity being highly skewed (Figure 4B). Following 2019, the annual count of the top 10 most productive scholars in the field exhibited a marked increase (Figure 4A). LI YANG topped the list with 34 publications, and their H-index (20) and G-index (34) were the highest among all authors, underscoring their substantial academic influence in this field (Table 3).

**FIGURE 4:**
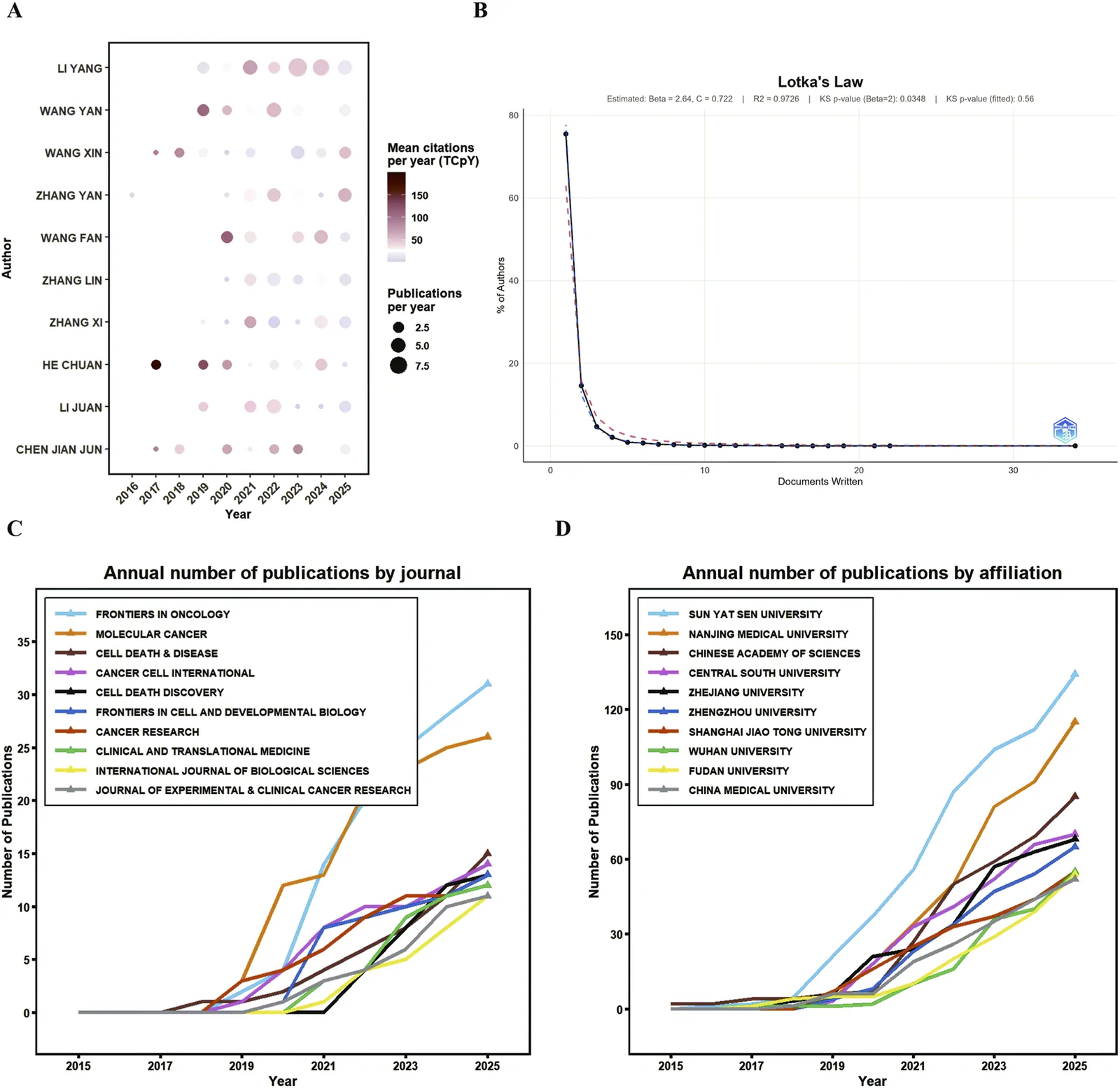
Productivity analysis of authors, journals, and institutions. **(A)** Annual publication output of the top ten most prolific authors. The size of the circle indicates the number of publications in the current year. The color scale (from light red to deep red) indicates the average number of citations (TCpY) per year. **(B)** Distribution of authors’ publication output based on Lotka’s Law. The x-axis represents the number of documents written, and the y-axis shows the percentage of corresponding authors. **(C)** Annual output of the top ten most productive journals. **(D)** Annual output of the top ten most productive institutions.

**TABLE 3:** The top 10 authors by number of publications.

Author	h_index	g_index	m_index	TC	NP	PY_start
LI YANG	20	34	2.50	1247	34	2019
WANG YAN	16	22	2.00	1939	22	2019
WANG XIN	15	21	1.50	2272	21	2017
ZHANG YAN	14	19	1.27	730	19	2016
WANG FAN	12	18	1.71	1513	18	2020
ZHANG LIN	11	17	1.57	438	17	2020
ZHANG XI	11	16	1.38	743	16	2019
HE CHUAN	13	15	1.30	4058	15	2017
LI JUAN	9	15	1.13	854	15	2019
CHEN JIAN JUN	12	12	1.20	2720	12	2017

At the journal level, the annual publication output has increased steadily year by year (Figure 4C). Frontiers in Oncology (the line indicated by the blue arrow) is particularly prominent, ranking first with 31 articles and recording an H-index of 17, a G-index of 29, and 862 total citations. It is followed by Molecular Cancer with 26 articles and the highest H-index (25) (Table 4). Regarding research institutions, Sun Yat-sen University and Nanjing Medical University are among the most productive. The annual publication volume of these institutions has maintained steady growth, especially since 2019 (Figure 4D).

**TABLE 4:** The top ten core journals in which m^6^A demethylase published papers in the field of tumor research.

Source	h_index	g_index	m_index	TC	NP	PY_start
FRONTIERS IN ONCOLOGY	17	29	2.13	862	31	2019
MOLECULAR CANCER	25	26	3.13	6016	26	2019
CELL DEATH and DISEASE	12	15	1.33	1030	15	2018
CANCER CELL INTERNATIONAL	10	14	1.25	982	14	2019
CELL DEATH DISCOVERY	11	13	2.20	309	13	2022
FRONTIERS IN CELL AND DEVELOPMENTAL BIOLOGY	10	13	1.43	260	13	2020
CANCER RESEARCH	11	12	1.38	2035	12	2019
CLINICAL AND TRANSLATIONAL MEDICINE	11	12	1.83	407	12	2021
JOURNAL OF EXPERIMENTAL and CLINICAL CANCER RESEARCH	10	11	1.43	785	11	2020
ONCOGENE	10	11	1.25	673	11	2019

### 3.4 Collaboration network among authors, institutions and countries

Based on publication counts, this study constructed collaboration networks across three dimensions: authors, institutions, and countries/regions, using Total Link Strength (TLS) as a quantitative metric to assess collaboration intensity among these entities. The author collaboration network (Figure 5A) visualizes co-authorship patterns and publication outputs. Applying a minimum publication threshold of five articles per author, 38 authors were included in the network. The visualization reveals several core author collaboration clusters, with Wang Hongsheng, Li Jiexin, and Chen Feng serving as central nodes, thereby constituting key hubs within the author collaboration network in this field.The institutional collaboration network (Figure 5B) illustrates the collaborative connections and the distribution of TLS among core research institutions. Applying a minimum publication threshold of 14 articles per institution, 23 institutions were included in the network. In the network, node sizes represent publication volumes, line thickness indicates collaboration intensity, and different colors distinguish institutional collaboration clusters. The top three institutions in terms of TLS were Sun Yat-sen University (44), the Chinese Academy of Sciences (27), and Nanjing Medical University (23).The country/region co-authorship network (Figure 5C) reflects the landscape of international scientific collaboration. Applying a minimum publication threshold of three articles per country/region, 15 countries were included in the network. In this network, the top two countries ranked by TLS-China (97) and the United States (92)-serve as the two core nodes of the international collaboration network in this field. The most prominent line thickness between these two countries reflects their strong collaborative ties and high frequency of interaction within this research area.

**FIGURE 5:**
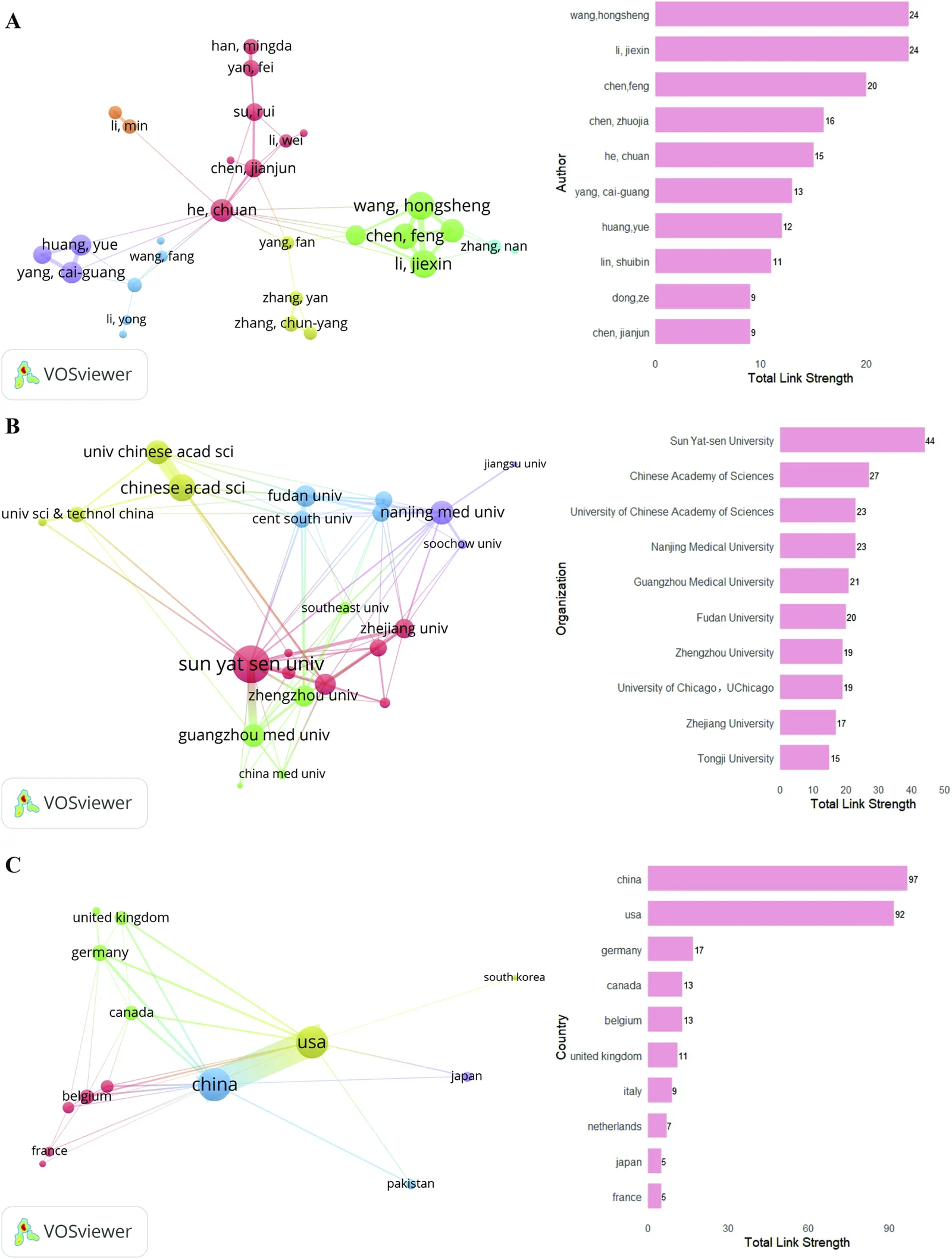
Collaboration networks among authors, institutions, and countries/regions. **(A)** Author collaboration network. Nodes represent authors; Node size indicates published output; Links represent co-authorship relationships; The link thickness reflects TLS, and the publication count threshold is ≥ 5. **(B)** Institutional collaboration network. Node representative organization; Node size represents the published output; Links represent inter-agency cooperation; The link thickness reflects TLS, and the publication count threshold is ≥ 14. **(C)** Country/region collaboration network. Nodes represent countries; Node size represents publication output; Links represent international co-authors; The link thickness reflects the total number of links and the publication count threshold ≥3.

### 3.5 Research hotspots and Frontier evolution based on keywords

Keywords serve as the core summary of a research article. The top 20 high-frequency keywords are listed in Table 5, including subject keywords such as “RNA Methylation” (45), “m^6^A Modification” (27), and “N6-methyladenosine” (23); disease keywords such as “gastric cancer” (21), “hepatocellular cancer” (21), “colorectal cancer” (20), and “breast cancer” (19); and other keywords such as “tumor microenvironment” (14) and “cancer therapy” (9). These results indicate that gastric cancer, liver cancer, and colorectal cancer are the most extensively studied cancer types in the context of m^6^A demethylase research, with their immune microenvironments and therapeutic interventions representing the primary research foci in this field. The keyword co-occurrence network further corroborates the above findings (Figure 6E).

**TABLE 5:** The 20 most commonly used keywords.

Rank	Words	Occurrences	Rank	Words	Occurrences
1	rna methylation	45	11	m6a methylation	13
2	m6a modification	27	12	m (6)a methylation	11
3	n6-methyladenosine (m6a)	23	13	Lung cancer	10
4	Gastric cancer	21	14	Pancreatic cancer	10
5	Hepatocellular carcinoma	21	15	Acute myeloid leukemia	10
6	Colorectal cancer	20	16	Cancer therapy	9
7	Breast cancer	19	17	Ovarian cancer	9
8	m (6)a modification	18	18	Bladder cancer	7
9	rna modification	18	19	Cell cycle	7
10	Tumor microenvironment	14	20	m6a demethylase	7

**FIGURE 6:**
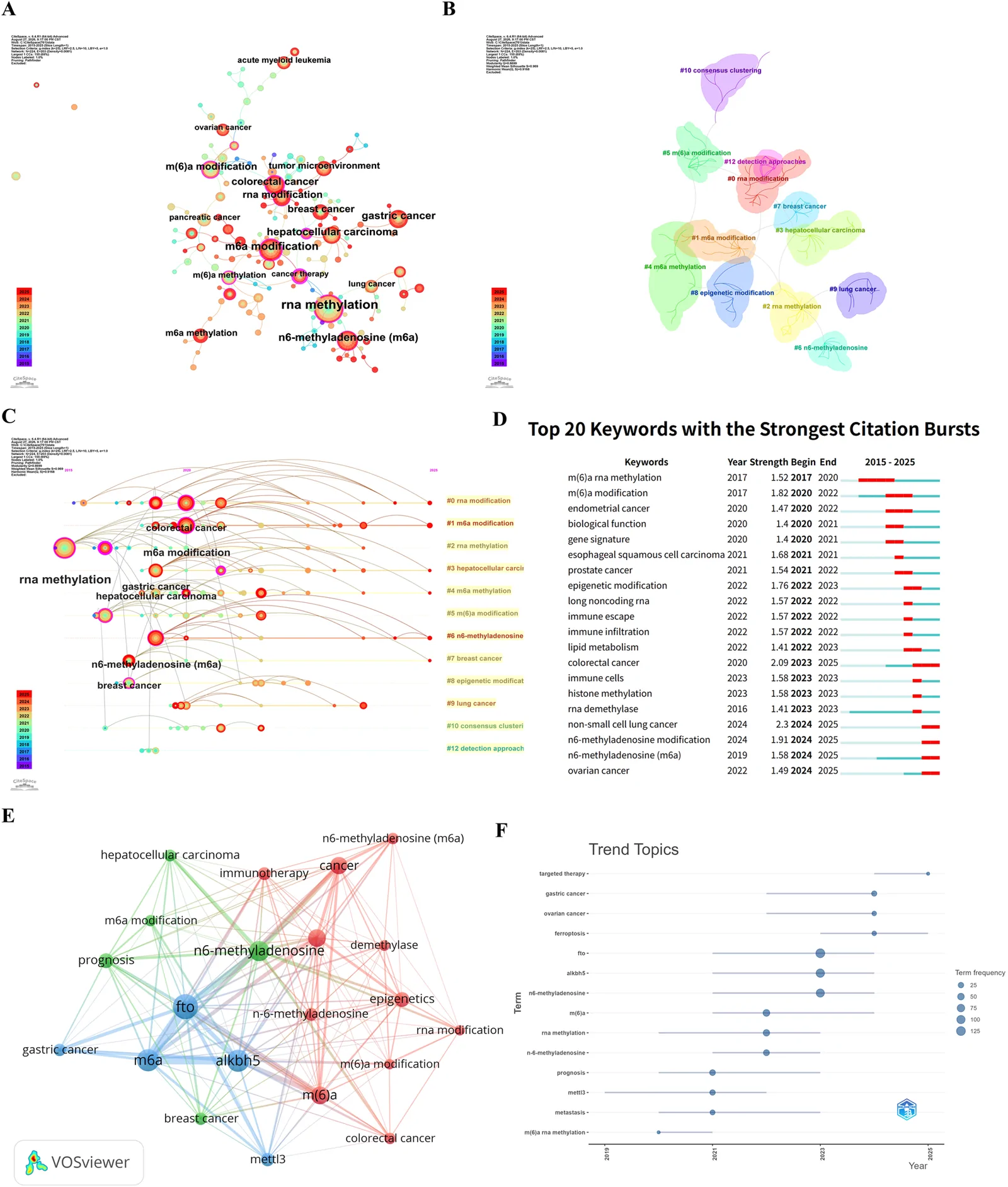
Multidimensional analysis based on keywords. **(A)** Keyword co-occurrence network. Nodes represent keywords; Node size indicates the frequency of keywords; Links represent co-occurrence relationships. **(B)** Keyword cluster diagram. **(C)** Keyword timeline view. **(D)** Visualization of top 20 keywords with the strongest citation bursts. Red Bars indicate burst duration; “Strength” indicates burst intensity. **(E)** keyword collaboration network. Nodes represent keywords; Node size indicates the frequency of occurrence; The line thickness represents the Total Link Strength, and the threshold is ≥ 18. **(F)** Keyword time-zone view. Terms represent high-frequency keywords; bubble size indicates frequency within each time slice; placement reflects temporal evolution of topic emphasis.Word Minimum Frequency: 5; Number of Words per Year: 2.

Keyword co-occurrence analysis (Figure 6A) facilitates the identification of semantic relationships among keywords and extracts latent information from textual data, thereby enhancing the understanding of research content and associated topics. To ensure analytical accuracy, keywords with closely related semantics were clustered and integrated, ultimately yielding 13 major clusters (Figure 6B). These clusters were primarily categorized into three groups: thematic clusters, cancer-type clusters, and epigenetic modification clusters. Based on these clusters, a timeline graph of keywords was constructed (Figure 6C), which illustrates the evolutionary trajectory and temporal distribution of keywords within each cluster. The modularity Q value (Q) and the weighted mean silhouette value (S) were used to evaluate the rationality of the network structure. In this study, Q = 0.8699 (>0.3) and S = 0.969 (>0.7), indicating a significant clustering structure and reliable results. As shown in the visualization map (Figure 6D) depicting the top 20 keywords with the strongest citation bursts, “non-small cell lung cancer” exhibited the highest burst strength (2.3). The temporal evolution of this research field can be divided into distinct developmental stages (Figure 6F), shifting from targeted therapy across various cancer types in the early phase to the identification of diverse m^6^A demethylases in the later phase, with research foci gradually transitioning toward tumor metastasis and prognosis evaluation.

### 3.6 Citation dynamics and paradigm shift in this field

Citation analysis reveals the evolving knowledge landscape in this field. The most frequently cited document is the review “The Role of m^6^A Modification in Biological Functions and Diseases” published by Xiulin Jiang’s team in Signal Transduction and Targeted Therapy in 2021. The top 20 cited publications were published in high-impact journals, and their total citation counts all exceeded 680 (Figure 7A; Table 6). In the citation burst analysis of the top 30 references (Figure 7B), the article exhibiting the highest burst intensity (25.26) discusses how, under hypoxic conditions, HIF-dependent signaling induces the expression of the m^6^A demethylase ALKBH5, which demethylates NANOG mRNA and promotes its stable expression, thereby facilitating the enrichment of the breast cancer stem cell phenotype (Zhang et al., 2016).

**FIGURE 7:**
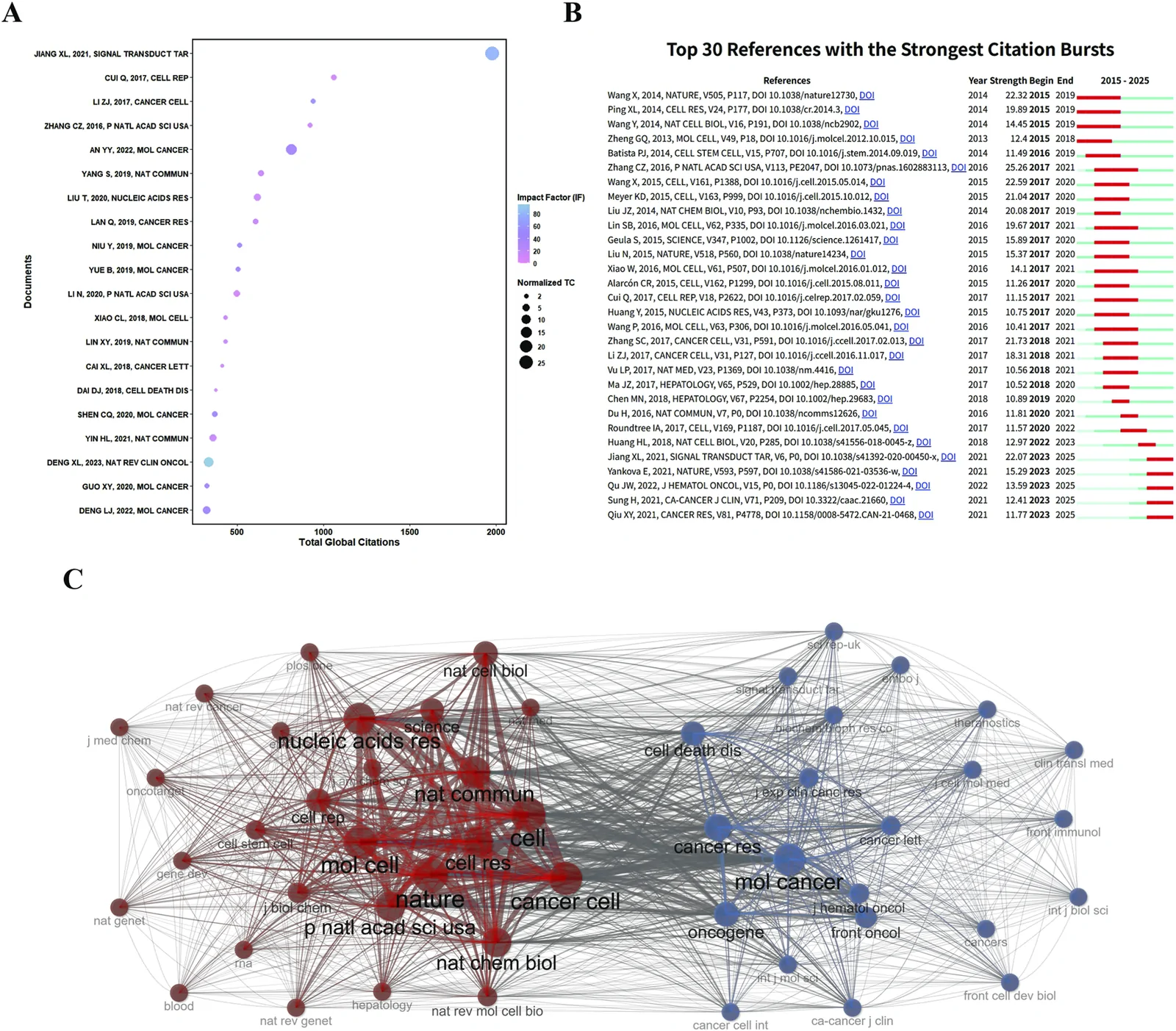
Citation analysis. **(A)** Bubble chart of the 20 most cited publications. Bubble size represents total citations; x-axis represents total Global Citations; y-axis represents papers, color represents journal impact factor. **(B)** Visualization of top 30 publications with the strongest citation bursts. Red Bars indicate burst duration; “Strength” indicates burst intensity. **(C)** Journal co-citation network.

**TABLE 6:** The top 10 most cited publications.

Paper	DOI	Total Citations	TC per Year	Normalized TC
JIANG XL, 2021, SIGNAL TRANSDUCT TAR	10.1038/s41392-020-00450-x	1978	329.7	27.15
CUI Q, 2017, CELL REP	10.1016/j.celrep.2017.02.059	1058	105.8	2.77
LI ZJ, 2017, CANCER CELL	10.1016/j.ccell.2016.11.017	940	94	2.46
ZHANG CZ, 2016, P NATL ACAD SCI USA	10.1073/pnas.1602883113	922	83.8	2.59
AN YY, 2022, MOL CANCER	10.1186/s12943-022-01500-4	814	162.8	15.44
YANG S, 2019, NAT COMMUN	10.1038/s41467-019-10669-0	640	80	3.37
LIU T, 2020, NUCLEIC ACIDS RES	10.1093/Nar/gkaa048	619	88.4	5.10
LAN Q, 2019, CANCER RES	10.1158/0008-5472.CAN-18-2965	605	75.6	3.18
NIU Y, 2019, MOL CANCER	10.1186/s12943-019-1004-4	513	64.1	2.70
YUE B, 2019, MOL CANCER	10.1186/s12943-019-1065-4	506	63.2	2.66

The journal co-citation analysis revealed a core clustering structure of authoritative journals characterized by interdisciplinary integration. The co-cited journals were mainly concentrated in leading international comprehensive and specialized journals such as Cell, Nature, and Molecular Cancer (Figure 7C), underscoring the high authority of research outputs in this field and the trend toward interdisciplinary convergence.

### 3.7 The evolution track of the research topic

To systematically analyze the evolution of research hotspots regarding m^6^A demethylases in the field of tumor research, this study employed multiple correspondence analysis to generate a conceptual structure map based on author keywords and indexed keywords, and plotted keyword clusters within a two-dimensional diagram (Figure 8A). The first cluster, marked in blue, mainly comprises common solid tumors such as gastric cancer and lung cancer, and is associated with the tumor microenvironment, immunotherapy, and prognosis. This reflects the heterogeneity of m^6^A modification across different cancer types and its critical role in immune microenvironment remodeling and clinical prognosis. The second cluster, marked in green, focuses on RNA modifications and epitranscriptomics, representing current and future research frontiers. The third cluster, marked in red, shows methyltransferases and demethylases plotted separately in the lower right corner, highlighting the significance of targeting m^6^A regulatory enzymes as emerging anticancer strategies. Topic analysis was performed using author keywords and their interrelationships to identify research themes (Figure 8B). Immunotherapy and the tumor microenvironment were identified as Motor Themes, indicating that these topics are both central and well-developed.

**FIGURE 8:**
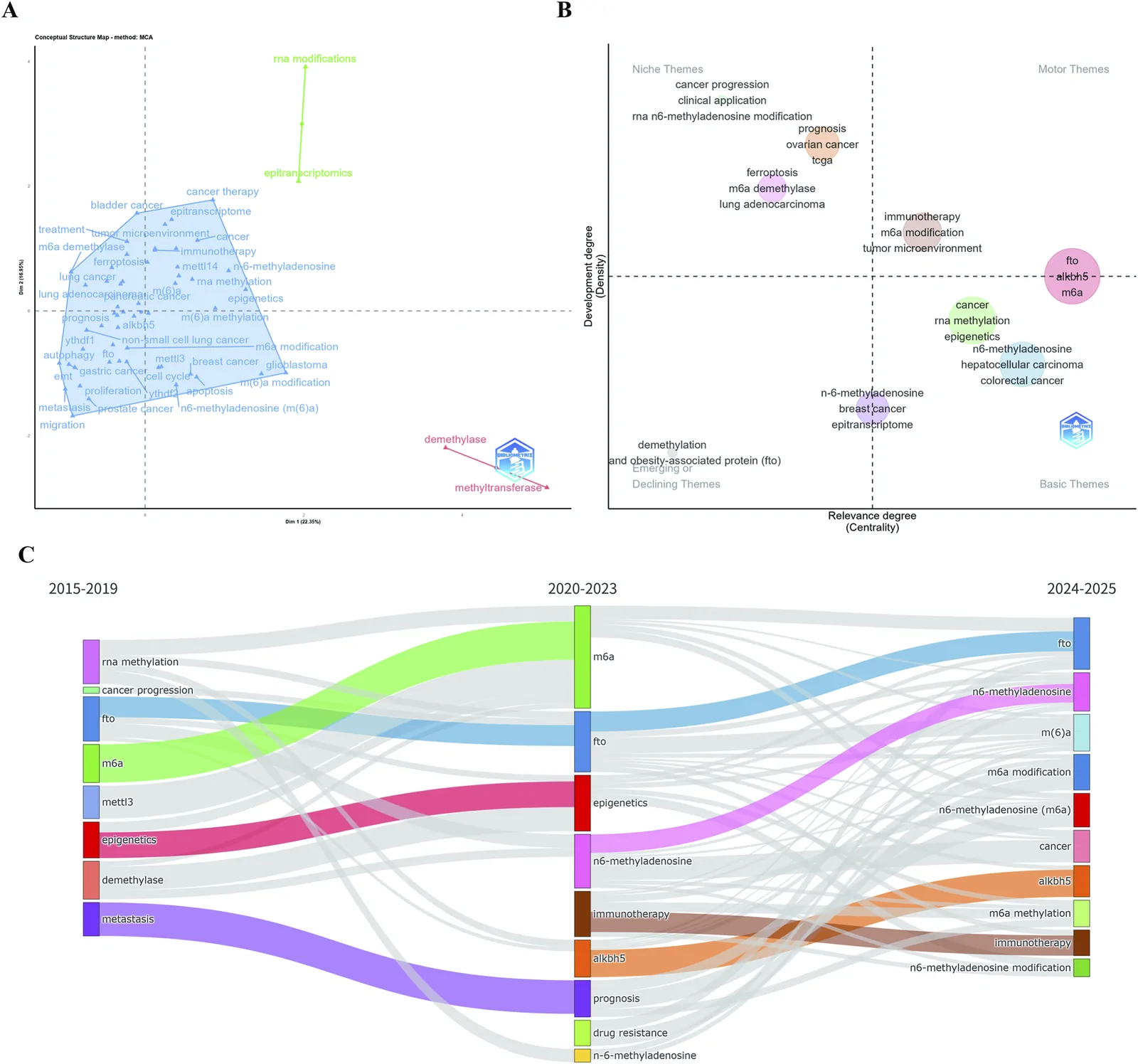
Thematic evolution analysis. **(A)** Multiple Correspondence Analysis strategic map. Points represent keywords; proximity indicates conceptual similarity based on co-occurrence. **(B)** Thematic map. Density is represented by the vertical axis, while centrality is represented by the horizontal axis. Centrality is the degree of correlation between different themes; Density measures the cohesion between nodes. **(C)** Thematic evolution map. Themes are tracked across time slices; links indicate how themes evolve/merge/split between periods. extract the top 250 terms; require a minimum frequency of five occurrences per 1,000 documents for each cluster; for visualization, display three labels per cluster with label size set to 0.3; construct the network using the Louvain clustering algorithm; and set the community repulsion parameter to 0.5.

The topic evolution diagram illustrates the temporal distribution and evolutionary trajectory of research themes from 2015 to 2025 (Figure 8C). From 2015 to 2019, research primarily focused on RNA methylation, FTO, m6A, METTL3, demethylases, genetics, as well as cancer progression and metastasis. This indicates that early studies were mainly devoted to elucidating the “writer-eraser” mechanism of m^6^A and its preliminary regulatory effects on the malignant phenotypes of tumor cells. From 2020 to 2023, with the deepening of research, m^6^A and FTO remained central, and were closely associated with N^6^-methyladenosine, genetics, ALKBH5, and immunotherapy. Notably, prognosis and drug resistance emerged as key focal points during this stage, reflecting a transition in research focus from single basic mechanisms to clinicopathologic features and oncologic treatment challenges (e.g., immune escape and drug resistance). From 2024 to 2025, Frontier trends indicate that research will further advance toward clinical translation and precision medicine. Most prominently, the sustained emergence and increasing prominence of immunotherapy suggest that future m^6^A research will prioritize combined strategies involving targeted regulatory enzymes, tumor immunotherapy, and epigenetic precision medicine.

### 3.8 Cross-database sensitivity analysis based on PubMed data

PubMed and WoSCC are not independent samples, so this part does not constitute independent verification, but only serves as a cross-database sensitivity analysis to test whether the main results of WoSCC change with the database replacement. The results show that the annual number of publications in this field has been increasing, which is largely consistent with the overall growth trend observed in the WoSCC dataset (Figure 9A). At the national level, China and the United States remained the top two contributors (Figure 9B). In terms of journal distribution, Frontiers in Oncology ranked first with 24 articles (Figure 9C). Multiple correspondence analysis (Figure 9D) revealed three distinct keyword clusters. The first cluster, marked in red, focuses on basic experimental research. Its core nodes include mice, animals, nude mice, and cell lines, indicating that *in vitro* and *in vivo* experimental models are the primary research approaches in this field. The second cluster, marked in blue, is oriented toward clinical samples and epigenetic regulation. Core nodes include humans, methylation, epigenetics, and messenger RNA. The third cluster, marked in green, features an independent prognosis node, reflecting an independent thematic direction that ultimately extends toward clinical translation and prognosis evaluation. To sum up, PubMed sensitivity analysis is consistent with the results of WoSCC in terms of annual trend, national distribution and theme structure. However, in view of the high degree of overlap between the two databases and the fact that this study has not been included in Scopus, Embase and other databases, the results should be used as a reminder of consistency.

**FIGURE 9:**
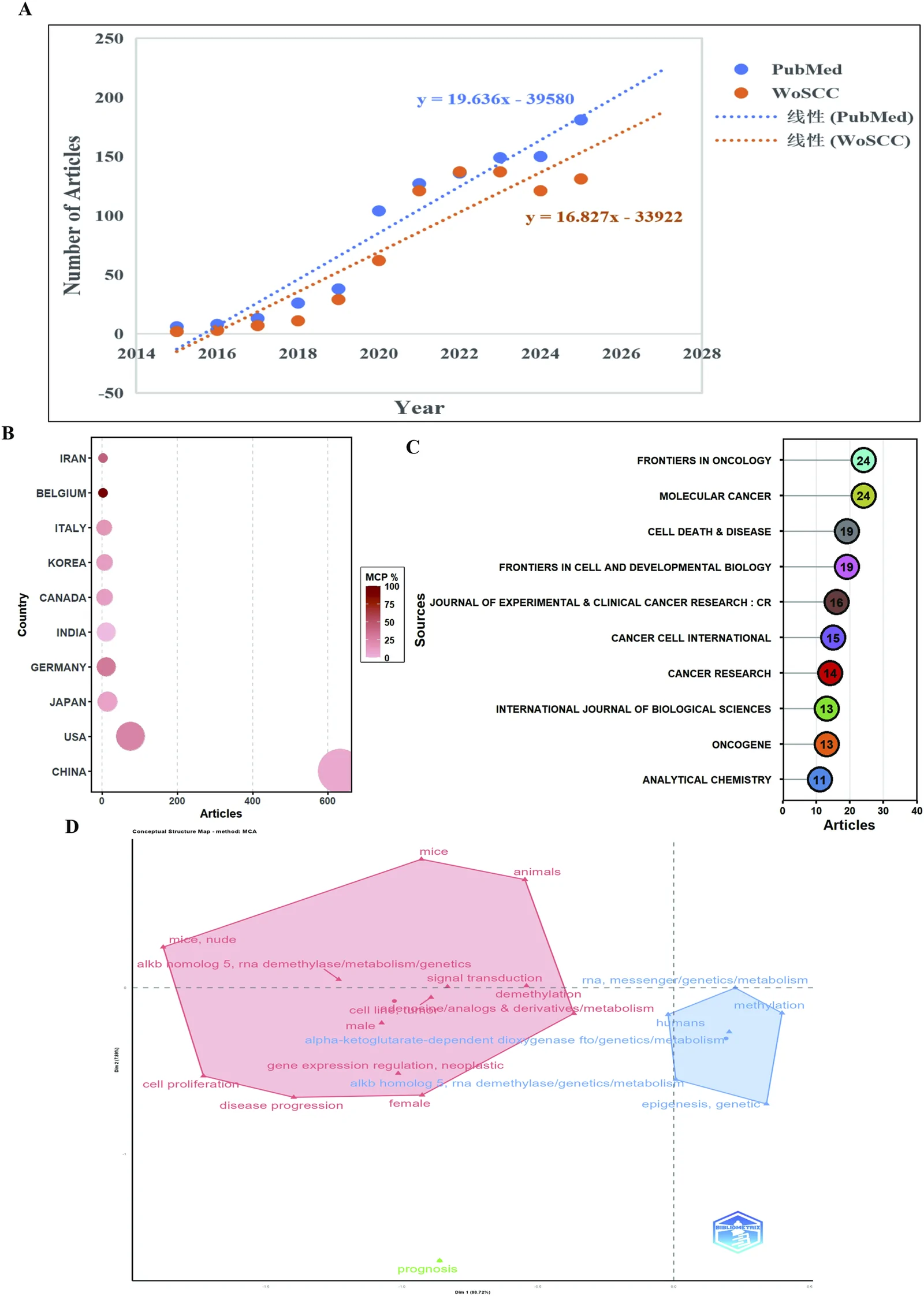
Cross-database sensitivity analysis based on PubMed data **(A)** Annual publication trends. **(B)** Bubble chart of the top ten countries by publication volume. **(C)** Lollipop chart of the top ten most productive journals. **(D)** Multiple Correspondence Analysis plot. Extract the top 250 terms; require a minimum frequency of five occurrences per 1,000 documents for each cluster; calculate weights using the “inclusion index weighted by term frequency”; and set the minimum weight-index threshold to 0.1.

## 4 Discussion

In this study, bibliometric and visual analysis methods were employed to systematically map the global knowledge landscape of m^6^A demethylases and tumors. The results demonstrate that this field has entered a phase of rapid growth since 2020, with an average annual growth rate exceeding 50%. It has emerged as an active area of investigation at the intersection of epigenetics and cancer research, exhibiting a developmental trend characterized by in-depth exploration of basic mechanisms, accelerating clinical translation, and deep multidisciplinary integration. In terms of the research landscape, China ranks first globally in total publication output, with institutions such as Sun Yat-sen University and Nanjing Medical University forming prominent research clusters, highlighting their substantial research capabilities and domestic innovation potential. Meanwhile, countries such as the United Kingdom and Belgium occupy central positions in the international collaboration network and the output of high-impact publications, contributing to a global research system marked by complementary cooperation between Eastern and Western regions. A stable collaborative network of highly productive scholars and core institutions has been established in this field; however, the overall distribution of researchers remains dispersed, leaving considerable room for further expansion. Analysis of the research trajectory and thematic evolution reveals that knowledge flow in this field radiates from molecular biology to genetics, clinical medicine, and materials science. The research focus has evolved from the early characterization of basic mechanisms involving FTO and ALKBH5 to the clinical translation level, encompassing the tumor immune microenvironment, therapeutic resistance, and prognosis evaluation. Notably, immunotherapy and the tumor microenvironment were identified as Motor Themes, suggesting that precision medicine strategies targeting m^6^A regulatory enzymes in combination with immune checkpoint blockade will represent a prominent focus of future research.

### 4.1 Concept traceability and foundation period

FTO was first identified as a obesity-susceptibility gene identified through genome-wide association studies, and later confirmed as a member of the Fe(II) and 2- ketoglutarate-dependent dioxygenase family, which can erase the N6 methyl group of RNA adenine, and is the first identified m^6^A demethylase (Azzam et al., 2022; Marcinkowski et al., 2021). This discovery redefines m^6^A from a static apparent marker to a reversible dynamic regulatory layer. From 2015 to 2020, the concept of reversible regulation of m^6^A rapidly extended to oncology, and FTO promoted the progress of acute myeloid leukemia by removing m^6^A modification on MYC, CEBPA and other transcripts (Li et al., 2017); On the other hand, ALKBH5 showed a selective cancer-promoting effect in AML, and its high expression suggested a poor prognosis (Shen et al., 2020)^.^ However, the functional verification in solid tumors is gradually spread out: ALKBH5 maintains the tumorigenicity of glioblastoma stem cells (Zhang et al., 2017); FTO not only enhances the tumorigenicity of melanoma, mediates the resistance to PD-1 therapy (Yang et al., 2019), but also drives the progression of breast cancer by inhibiting BNIP3 (Niu et al., 2019). In the aspect of treatment transformation: FTO targeted intervention represented by R-2HG has shown anti-tumor activity (Su et al., 2018), suggesting that “eraser” has the potential to become a drug. Overall, the amount of papers published at this stage only accounts for 6.83%. The research content is mainly about molecular identification and function of demethylase.

### 4.2 Peak period: prognostic value map of m^6^A demethylases based on cancer types with high incidence

Since 2020, the number of publications in this field has increased rapidly, and the research focus has gradually shifted from exploring the basic functions of FTO to the regulation of tumor prognosis, invasion, and metastasis. Gastric cancer, hepatocellular carcinoma, and colorectal cancer have emerged as the core research subjects, which correspond to the medium- and high-frequency disease keywords identified in the analysis. During this stage, studies revealed remarkable cancer-type-specific effects of m^6^A demethylases. In gastric cancer, FTO is highly expressed and promotes tumorigenesis and metastasis by mediating m^6^A modification and mitochondrial dynamic reprogramming of caveolin-1 (Zhou et al., 2022). In hepatocellular carcinoma, downregulation of FTO expression reduces m^6^A demethylase activity, leading to an abnormal increase in m^6^A modification levels of specific oncogenes, which subsequently enhances the invasion and metastasis of hepatocellular carcinoma cells by regulating related signaling pathways (Zhou et al., 2025). Additionally, hypoxia microenvironment-mediated downregulation of FTO has been found to promote tumor metastasis in colorectal cancer (Ruan et al., 2021). Meanwhile, ALKBH5 also exhibits dual regulatory effects. In hepatocellular carcinoma, ALKBH5 post-transcriptionally inhibits the oncogene LYPD1 via m^6^A demethylation, thereby suppressing the proliferation, invasion, and metastasis of hepatocellular carcinoma cells, suggesting that ALKBH5 is an independent prognostic factor for this cancer (Chen et al., 2020). In gastric cancer, ALKBH5 inhibits cancer cell invasion by mediating m^6^A demethylation of PKMYT1 (Hu et al., 2022). In contrast, in colorectal cancer, ALKBH5 stabilizes RAB5A mRNA through a YTHDF2-dependent pathway, subsequently activating RAB5A post-transcriptionally to promote tumor progression (Shen et al., 2023). During this phase, the majority of publications were contributed by Chinese scholars, and international collaborative research remained limited. This is consistent with the quantitative analysis showing that 89.75% of publications from China were SCP. This pattern may be related to factors such as domestic funding priorities, self-sufficiency of large research teams, and language barriers.

### 4.3 Frontier hotspots

#### 4.3.1 m^6^A demethylase in reshaping the tumor-immunosuppressive microenvironment

As a core epigenetic “molecular switch” reshaping the immunosuppressive tumor microenvironment, m^6^A demethylases have emerged as a prominent research focus in tumor immunology. In colorectal cancer, ALKBH5 is highly expressed and activates the Wnt/DKK1 signaling axis by mediating m^6^A demethylation and degradation of AXIN2 mRNA, thereby inducing an immunosuppressive microenvironment that is closely associated with poor patient prognosis. Targeting this pathway effectively reverses immune escape and enhances the efficacy of anti-PD-1 therapy (Zhai et al., 2023). ALKBH5 also regulates paracrine interactions between tumor cells and tumor-associated macrophages, driving the progression of non-small cell lung cancer and modulating sensitivity to anti-PD-L1 therapy (Hua et al., 2024). Meanwhile, fat mass and obesity-associated protein exerts key regulatory functions. Targeting the FTO/m6A/GPNMB axis inhibits the growth and metastasis of hepatocellular carcinoma and reshapes the immune activation state, highlighting the clinical translational potential of FTO small-molecule inhibitors (Chen et al., 2024). In breast cancer, the hypoxic microenvironment upregulates PD-L1 expression through an FTO-dependent m^6^A/YTHDF3/PDK1 pathway, ultimately mediating an immunosuppressive phenotype (Wang et al., 2025). Current research has clearly moved beyond the independent regulation of single tumor cells to focus on immune-related m^6^A modifications and remodeling, which is consistent with the molecular biology and immunology knowledge domain identified in the dual-map overlay analysis of this study.

#### 4.3.2 m^6^A demethylase-driven therapy for resistance

Overcoming acquired drug resistance remains a major challenge in the precision treatment and clinical translation of oncology. RNA demethylases play a pivotal role in the multi-level regulation of tumor targeted therapy resistance by reshaping the m^6^A modification landscape. FTO diminishes the sensitivity of colorectal cancer cells to 5-FU via the FTO–SIVA1 axis (Lin Z. et al., 2023), and promotes gemcitabine resistance in pancreatic cancer and osimertinib resistance in EGFR-mutant lung adenocarcinoma by reducing m^6^A modification levels (Lin K. et al., 2023; Chen J. et al., 2025). Concurrently, ALKBH5 mediates docetaxel resistance in prostate cancer by regulating CLSPN expression via the ALKBH5–IGF2BP2 axis (Chen Y. et al., 2025), and drives cetuximab resistance in KRAS-mutant colorectal cancer by activating FOXM1 through demethylation (Han et al., 2025); the resulting accumulation of 27HC due to its deletion serves as a microenvironmental basis for lenvatinib resistance and immune escape in hepatocellular carcinoma (Wang et al., 2026). Targeting m^6^A demethylases and their downstream effector molecules represents a potential strategy to reverse tumor drug resistance.

### 4.4 Future prospects

Based on the evolutionary trends of this field, the global research landscape, and existing knowledge gaps-and integrating the latest advances in translational medicine from the past three years-future research should prioritize three strategic directions: clinical translation, international collaboration, and multidisciplinary integration. First, efforts should be directed toward promoting multi-level clinical translation and establishing a comprehensive precision diagnosis and treatment system. There is an urgent need to conduct multicenter prospective cohort studies to construct tumor recurrence prediction models based on m^6^A modification profiles (Xu X. et al., 2025), thereby providing systematic evidence-based support for RNA methylation as a liquid biopsy biomarker and bridging the gap from basic discovery to clinical application (Xu C. et al., 2025). Second, given the imbalanced pattern in which China leads in publication volume but the international collaboration rate remains low at only 10.25%, it is essential to strengthen global multicenter research cooperation, balance regional research development, and establish standardized public multi-omics data repositories. Third, deepening interdisciplinary integration will expand the boundaries of innovation. By moving beyond a single-discipline paradigm through the integration of materials science (targeted nano-delivery), artificial intelligence (AI; m^6^A prognosis algorithms), and immunology (single-cell microenvironment mapping), we can advance the core objective of serving precision oncology.

### 4.5 Advantages and limitations

In this study, bibliometric analysis was employed to examine the knowledge structure in this field from a quantitative and macroscopic perspective, thereby revealing the developmental trends and research hotspots of m^6^A demethylases in tumor research. Through multidimensional systematic analysis, this study provides in-depth references for related research. However, several limitations should be acknowledged. The discrepancies observed between the WoSCC and PubMed datasets in terms of journal counts and national publication outputs are primarily attributable to differences in their collection scopes and statistical criteria. WoSCC encompasses all types of academic publications (e.g., research articles and reviews), whereas PubMed places greater emphasis on the screening of clinical medical literature, resulting in partial heterogeneity in the included documents. Because PubMed and WoSCC are highly overlapped, there is no formal cross-database consistency statistical test in this study, and the results of sensitivity analysis should be interpreted descriptively. Furthermore, this study has several limitations, including language restrictions; the exclusion of other databases such as Scopus; citation-timing biases; database indexing discrepancies; dependence on software parameters; potential inaccuracies arising from keyword synonyms; the influence of self-citations; limitations of aggregate statistical methods; and the inability of bibliometric approaches to evaluate experimental quality. Additionally, potential biases inherent in the bibliometric algorithms based on natural language processing may also affect the analytical results.

## 5 Conclusion

In this study, the research landscape of m^6^A demethylases in the field of oncology was delineated through a bibliometric panorama. This field has experienced rapid growth since 2020. Although China holds a dominant position in publication output, the breadth of international collaboration remains to be further expanded. The research focus has evolved from the early identification of basic mechanisms to the tumor immune microenvironment, therapeutic resistance, and prognosis evaluation. In the future, efforts should be directed toward breaking down disciplinary barriers, deepening international cooperation, and facilitating advances in the precision diagnosis and treatment of tumors.

## Data Availability

The original contributions presented in the study are included in the article/Supplementary Material, further inquiries can be directed to the corresponding authors.
