# Supplementary material for: Global research trends in m6A demethylases and cancer: a bibliometric and visualization analysis
Source: Front Genet. 2026 Oct 7;17:1943884. doi: 10.3389/fgene.2026.1943884 (PMC13683196; doi:10.3389/fgene.2026.1943884)
Supplement: Supplementary file 1 [file Table1.docx]

MeSH result-Neoplasms

1: Neoplasms

New abnormal growth of tissue. Malignant neoplasms show a greater degree of

anaplasia and have the properties of invasion and metastasis, compared to benign

neoplasms.

Year introduced: /diagnosis was NEOPLASM DIAGNOSIS 1964-1965

Subheadings:

blood

blood supply

cerebrospinal fluid

chemically induced

chemistry

classification

complications

congenital

diagnosis

diagnostic imaging

diet therapy

drug therapy

economics

embryology

enzymology

epidemiology

ethnology

etiology

genetics

history

immunology

metabolism

microbiology

mortality

nursing

parasitology

pathology

physiopathology

prevention and control

psychology

radiotherapy

rehabilitation

surgery

therapy

ultrastructure

urine

veterinary

virology

Tree Number(s): C04

Entry Terms:

Tumors

Neoplasia

Neoplasias

Neoplasm

Tumor

Cancer

Cancers

Malignant Neoplasm

Malignancy

Malignancies

Malignant Neoplasms

Neoplasm, Malignant

Neoplasms, Malignant

Benign Neoplasms

Neoplasms, Benign

Neoplasm, Benign

Benign Neoplasm

All MeSH Categories

Diseases Category

Neoplasms

Cysts

Arachnoid Cysts

Bone Cysts +

Branchioma

Breast Cyst

Bronchogenic Cyst

Chalazion

Choledochal Cyst

Colloid Cysts

Dermoid Cyst

Epidermal Cyst

Esophageal Cyst

Follicular Cyst

Ganglion Cysts

Lymphocele

Mediastinal Cyst

Mesenteric Cyst

Mucocele

Ovarian Cysts +

Pancreatic Cyst +

Parovarian Cyst

Pilonidal Sinus

Ranula

Synovial Cyst +

Tarlov Cysts

Thyroglossal Cyst

Urachal Cyst

Hamartoma

Hamartoma Syndrome, Multiple +

Pallister-Hall Syndrome

Tuberous Sclerosis

Neoplasms by Histologic Type

Histiocytic Disorders, Malignant +

Leukemia +

Lymphoma +

Neoplasm, Lymphatic Tissue +

Neoplasms, Complex and Mixed +

Neoplasms, Connective and Soft Tissue +

Neoplasms, Germ Cell and Embryonal +

Neoplasms, Glandular and Epithelial +

Neoplasms, Gonadal Tissue +

Neoplasms, Nerve Tissue +

Neoplasms, Plasma Cell +

Neoplasms, Vascular Tissue +

Nevi and Melanomas +

Odontogenic Tumors +

Neoplasms by Site

Abdominal Neoplasms +

Anal Gland Neoplasms

Bone Neoplasms +

Breast Neoplasms +

Digestive System Neoplasms +

Endocrine Gland Neoplasms +

Eye Neoplasms +

Head and Neck Neoplasms +

Hematologic Neoplasms +

Mammary Neoplasms, Animal +

Nervous System Neoplasms +

Pelvic Neoplasms

Skin Neoplasms +

Soft Tissue Neoplasms +

Splenic Neoplasms

Thoracic Neoplasms +

Urogenital Neoplasms +

Neoplasms, Experimental

Carcinoma 256, Walker

Carcinoma, Brown-Pearce

Carcinoma, Ehrlich Tumor

Carcinoma, Krebs 2

Carcinoma, Lewis Lung

Leukemia, Experimental +

Liver Neoplasms, Experimental

Mammary Neoplasms, Experimental

Melanoma, Experimental

Sarcoma, Experimental +

Neoplasms, Hormone-Dependent

Neoplasms, Multiple Primary

Hamartoma Syndrome, Multiple +

Multiple Endocrine Neoplasia +

Tuberous Sclerosis

Neoplasms, Post-Traumatic

Neoplasms, Radiation-Induced

Leukemia, Radiation-Induced

Neoplasms, Second Primary

Neoplastic Processes

Anaplasia

Carcinogenesis +

Neoplasm Invasiveness +

Neoplasm Metastasis +

Neoplasm Recurrence, Local

Neoplasm Regression, Spontaneous

Neoplasm, Residual

Oncogene Addiction

Neoplastic Syndromes, Hereditary

Adenomatous Polyposis Coli +

Basal Cell Nevus Syndrome

Birt-Hogg-Dube Syndrome

Colorectal Neoplasms, Hereditary Nonpolyposis +

Dysplastic Nevus Syndrome

Exostoses, Multiple Hereditary

Hamartoma Syndrome, Multiple

Hereditary Breast and Ovarian Cancer Syndrome

Li-Fraumeni Syndrome

Multiple Endocrine Neoplasia +

Neurofibromatoses +

Peutz-Jeghers Syndrome

Tuberous Sclerosis

Wilms Tumor +

Paraneoplastic Syndromes

Paraneoplastic Endocrine Syndromes +

Paraneoplastic Syndromes, Nervous System +

Paraneoplastic Syndromes, Ocular

Precancerous Conditions

Aberrant Crypt Foci

Barrett Esophagus

Erythroplasia

Keratosis, Actinic

Leukoplakia +

Lymphomatoid Granulomatosis

Preleukemia

Smoldering Multiple Myeloma

Uterine Cervical Dysplasia +

Xeroderma Pigmentosum

Pregnancy Complications, Neoplastic

Trophoblastic Neoplasms +
