# Supplementary material for: Global research trends in m6A demethylases and cancer: a bibliometric and visualization analysis
Source: Front Genet. 2026 Oct 7;17:1943884. doi: 10.3389/fgene.2026.1943884 (PMC13683196; doi:10.3389/fgene.2026.1943884)
Supplement: Supplementary file 2 [file Table2.docx]

**Table S2.** Retrieval strategy of publications in the study

| Citation index | |
| --- | --- |
| Web of Science Core Collection (WoSCC) | #TS1= ((TS=(m⁶A demethylase)) OR TS=(N⁶-methyladenosine demethylase)) OR TS=(m⁶A demethylating enzyme)  #TS2= ((((((((((((((((TS=(Tumors)) OR TS=(Neoplasia)) OR TS=(Neoplasias)) OR TS=(Neoplasm)) OR TS=(Tumor)) OR TS=(Cancer)) OR TS=(Cancers)) OR TS=(Malignant Neoplasm)) OR TS=(Malignancy)) OR TS=(Malignancies)) OR TS=(Malignant Neoplasms)) OR TS=(Neoplasm,Malignant)) OR TS=(Neoplasms, Malignant)) OR TS=(Benign Neoplasms)) OR TS=(Neoplasms, Benign)) OR TS=(Neoplasm, Benign)) OR TS=(Benign Neoplasm)  TS=TS1 AND TS2 |
| PubMed | #1= ((m^6^A demethylase) OR (N^6^-methyladenosine demethylase)) OR (m^6^A demethylating enzyme)  #2= ((((((((((((((((Tumor) OR (Neoplasia)) OR (Neoplasias)) OR (Neoplasm)) OR (Tumor)) OR (Cancer)) OR (Cancers)) OR (Malignant Neoplasm)) OR (Malignancy)) OR (Malignancies)) OR (Malignant Neoplasms)) OR (Neoplasm,Malignant)) OR (Neoplasms, Malignant)) OR (Benign Neoplasms)) OR (Neoplasms, Benign)) OR (Neoplasm, Benign)) OR (Benign Neoplasm)  #1 AND #2 |
| Document  type | Article OR Review Article |
| Language | English |
| Timespan | 2015.01.01-2025.12.31 |
